# Supplementary material for: Radiation Exposure from GEP NET Surveillance
Source: Cancers (Basel). 2024 Jan 19;16(2):427. doi: 10.3390/cancers16020427 (PMC10814752; doi:10.3390/cancers16020427)
Supplement: Supplementary file 1 [file cancers-16-00427-s001.zip › cancers-2832989-supplementary.pdf]

Supplementary Table S1: Imaging recommendations by different consensus guidelines for completely resected stage I to III small bowel NETs

**Guideline**	**Cross sectional imaging**		**Nuclear medicine imaging recommendation**	**Follow-up duration**
	**Modality**	**Frequency**		
NANETS	CT or MRI	Baseline at 6 months then annually	At baseline, then not recommended routinely	10 years
ENETS	CT or MRI	Every 6 to 12 months	SRS q 24 months	Not specified
NCCN	CT or MRI	Baseline at 3 to 12 months then every 12 to 24 months	No recommendation	10 years
ESMO	CT or MRI	Every 3 to 6 months	SRS after 12 to 36 months; frequency not specified	Lifelong
CommNETs	CT or MRI	Every 12 months for first three years, then every 12 to 24 months	No recommendation	10 years

NANETS: North American Neuroendocrine Tumor Society (2017)

ENETS: European Neuroendocrine Tumor Society (2017)

NCCN: National Comprehensive Cancer Network (2021)

ESMO: European Society for Medical Oncology (2020)

CommNETs: Commonwealth Neuroendocrine Tumor research collaborative (2018)

Supplementary Table S2. Cumulative radiation exposure associated with imaging protocols derived from different consensus guidelines.

Guideline	Imaging protocol permutations	CT mean dose (mSv)*	Number of CT scans	Number of 68Ga-DOTATATE PET scans **	Total effective dose (mSv)
NANETS	CT only	16.9	11	0	185.9
	Alternating CT and MRI	16.9	5.5	0	92.95
	MRI only	N/A	0	0	0
ENETS	CT only	16.9	12	5	272.05
	Alternating CT and MRI	16.9	6	5	170.65
	MRI only	N/A	0	5	69.25
NCCN	CT only	16.9	7	0	118.3
	Alternating CT and MRI	16.9	3.5	0	59.15
	MRI only	N/A	0	0	0
ESMO	CT only	16.9	20	2	365.7
	Alternating CT and MRI	16.9	10	2	196.7
	MRI only	N/A	0	2	27.7
COMMNET	CT only	16.9	7	0	118.3
	Alternating CT and MRI	16.9	3.5	0	59.15
	MRI only	N/A	0	0	0

NANETS: North American Neuroendocrine Tumor Society (2017)
ENETS: European Neuroendocrine Tumor Society (2017)
NCCN: National Comprehensive Cancer Network (2021)
ESMO: European Society for Medical Oncology (2020)
COMMNET: Commonwealth Neuroendocrine Tumor Research Collaboration (2018)

*CT mean dose (mSv) as calculated from our study
**68Ga-DOTATATE PET scan mean dose calculated from our study was 13.85mSv (includes CT component of scan)
